# Deep Learning-Based Prediction of Epithelial Cytokine Responses for the Selection of Functionally Consistent Airway Organoids

**DOI:** 10.3390/biomimetics11080547

**Published:** 2026-08-03

**Authors:** Hyeokjin Kweon, Mi Hyun Lim, David W. Jang, Keonhyeok Park, Seungchul Lee, Do Hyun Kim

**Affiliations:** 1 Department of Mechanical Engineering, Korea Advanced Institute of Science and Technology (KAIST), Daejeon 34141, Republic of Korea; leonkweon@kaist.ac.kr; 2 Department of Otolaryngology–Head and Neck Surgery, Seoul St. Mary’s Hospital, College of Medicine, The Catholic University of Korea, Seoul 06591, Republic of Korea; mhlim@catholic.ac.kr; 3 Department of Head and Neck Surgery & Communication Sciences, Duke University School of Medicine, Durham, NC 27710, USA; david.jang@duke.edu; 4 Department of Mechanical Engineering, Pohang University of Science and Technology (POSTECH), Pohang 37673, Republic of Korea; khpark0409@postech.ac.kr

**Keywords:** airway organoids, inflammatory cytokine prediction, bright-field image analysis, deep learning-based classification, texture feature analysis

## Abstract

Although airway organoids provide a physiologically relevant platform for modeling human airway inflammation, their utility is often limited by substantial heterogeneity in epithelial differentiation and functional responsiveness across Matrigel domes. Here, we present a non-destructive, imaging-guided framework to predict epithelial cytokine responses and enable the selection of functionally consistent airway organoid domes. Mature human airway organoids were stimulated with house dust mite (HDM) extract and dome-level inflammatory responsiveness was quantified by RT-qPCR for thymic stromal lymphopoietin (TSLP) and interleukin-33 (IL-33). Both cytokines exhibited wide dome-to-dome variability and showed a significant positive correlation, indicating coordinated allergic inflammatory regulation. Meanwhile, bright-field dome images were analyzed to segment individual organoids, define robust regions of interest, and extract quantitative morphological and texture descriptors based on gray-level co-occurrence matrix features. Organoid-level descriptors were aggregated into a single dome-level feature vector using distributional statistics, thereby capturing both central tendency and heterogeneity within each dome. Using these engineered dome-level features, we trained a deep tabular learning model (TabNet) to classify qPCR-defined inflammatory responsiveness. The resulting model achieved strong and consistent cross-validated performance for both targets, reaching balanced accuracies of 0.910 for TSLP and 0.833 for IL-33, demonstrating that bright-field phenotypes contain predictive signatures of cytokine activation. This approach provides a scalable enrichment strategy for robustly responsive organoid–Matrigel domes without destructive assay. It improves reproducibility in organoid-based airway inflammation studies and supports standardized dome selection for downstream mechanistic and translational applications.

## 1. Introduction

Human respiratory diseases impose a substantial health burden globally, with persistently high prevalence and morbidity rates [1,2]. However, the complex and heterogeneous pathophysiology of airway disorders and limitations inherent to conventional preclinical models remain challenging for further therapeutic advancements. Specifically, two-dimensional cell cultures fail to recapitulate the multicellular architecture and spatial organization of the human airway, while animal models often exhibit species-specific differences in immune responses and epithelial biology, leading to poor translational fidelity [3,4].

Organoid technology is a powerful platform that overcomes these limitations by enabling the generation of three-dimensional (3D) structures that recapitulate key cellular, structural, and functional features of human tissues [5,6,7]. Airway organoids, composed of diverse epithelial cell populations including basal, secretory, and ciliated cells, closely mimic the cellular composition and functional behavior of the native airway epithelium. Hence, they are particularly well suited for modeling airway inflammation and evaluating therapeutic responses [8,9]. Beyond airway models, bioengineered mucosal organoids derived from the upper aerodigestive tract—including nasal, oral, and maxillofacial mucosal epithelium—have emerged as versatile platforms for regenerative applications and preclinical screening [10,11]. Sinonasal epithelium, in particular, forms a continuous mucosal barrier that shares structural and immunological features with oral and maxillofacial mucosa, such as a pseudostratified epithelium, secretory cell populations, and barrier-associated alarmin responses [12].

To model these mucosal systems, airway organoids are commonly cultured within Matrigel-based dome structures that provide essential extracellular matrix support for differentiation and maturation [13]. However, a critical yet often overlooked challenge is the substantial heterogeneity that arises not only between individual organoids but also among organoids within the same dome. Because functional responses such as cytokine release following allergen exposure reflect the integrated behavior of an entire organoid, the variability of Matrigel complexes at the dome level can compromise reproducibility and limit the reliability of disease modeling.

To address this challenge, new strategies are needed to systematically identify organoid–Matrigel domes that exhibit robust and physiologically relevant inflammatory responses. Recent advances in deep learning-based object detection and segmentation have expanded the ability to extract quantitative information from bright-field Matrigel dome images [14,15,16,17]. Among these, multimodal attention-based detection frameworks have further advanced visual representation learning by incorporating multi-scale feature fusion and attention mechanisms [18,19,20]. However, airway organoid analysis differs fundamentally from conventional object detection: individual organoids are densely packed within each Matrigel dome and frequently overlap, making accurate boundary delineation substantially more demanding than object localization alone. Moreover, because our goal is to quantitatively characterize organoid morphology and internal texture rather than merely detect organoid locations, precise segmentation is better suited to extracting biologically relevant descriptors. Building on these segmentation-based approaches to precisely identify individual organoids and delineate their morphological structures, it is possible to analyze phenotyping descriptors such as size, brightness, and textures that can be directly linked to molecular readouts. This integration allows image-derived phenotypes to serve not only as descriptive morphological markers but also as predictive indicators of inflammatory activity. The biological basis for this approach lies in the close relationship between epithelial morphology and functional state. Because TSLP and IL-33 are epithelial alarmin cytokines released in response to barrier disruption and environmental stress, their induction is influenced by the differentiation state and structural organization of the airway epithelium [18]. In airway organoids, epithelial differentiation and maturation are reflected in morphological characteristics such as lumen architecture and epithelial folding [8]. Accordingly, quantitative features extracted from routine bright-field images may serve as surrogate indicators of epithelial functional state and provide biologically relevant information for predicting TSLP and IL-33 responsiveness following HDM stimulation.

In this study, we developed an integrated platform that combines bright-field deep learning analysis with quantitative PCR (qPCR) measurements of epithelial cytokines; these cytokines are released in response to house dust mite (HDM) stimulation. We trained deep learning models to predict cytokine expression from organoid images. Thus, we established a rapid and non-destructive method to identify Matrigel domes containing organoids with consistent and robust allergic inflammatory responses. This framework provides a scalable and standardized strategy for selecting organoid–Matrigel domes for downstream mechanistic and translational studies, thereby enhancing the fidelity of respiratory organoid models and broadening their applicability for drug screening and precision medicine. The main contributions of our work are summarized as follows:

- Imaging-guided quantification of dome-level heterogeneity. We introduce a robust image-analysis framework that quantifies intrinsic heterogeneity within Matrigel-based airway organoid domes by combining deep learning-based segmentation, geometry-constrained region of interest (ROI) refinement, and multi-scale texture feature extraction from standard bright-field images.
- Prediction of epithelial inflammatory responsiveness from morphology. We demonstrate that dome-level epithelial cytokine responses to HDM stimulation, defined by RT-qPCR measurements of TSLP and IL-33, can be accurately predicted using only image-derived morphological and textural descriptors through deep tabular learning.
- Non-destructive selection of functionally consistent airway organoids. We propose a scalable, non-destructive dome selection strategy that enables prospective enrichment of airway organoids with robust and consistent inflammatory responses, improving reproducibility and standardization in organoid-based airway inflammation models.

## 2. Materials and Methods

### 2.1. Airway Organoid Formation and Exposure to HDM

This study was approved by the Institutional Review Board of Seoul Saint Mary’s Hospital (KC18TESI0167), and written informed consent was obtained from all donors. All methods were performed in accordance with the relevant guidelines and regulations and the Declaration of Helsinki. Inferior turbinate tissue was collected during turbinectomy from two donors, rinsed in saline containing gentamicin, antibiotic–antimycotic solution, and phosphate-buffered saline (PBS; Welgene, Taipei, China), and minced into approximately $1\;{\mathrm{mm}}^{3}$ fragments. The tissue fragments were digested in 0.3% (*w*/*v*) collagenase (Gibco, Waltham, MA, USA) at 37 °C for 14–18 h and then passed through a 40μm cell strainer; the filtrate was then seeded into 100mm culture dishes containing Complete PneumaCult^TM^ Ex Plus medium (STEMCELL Technologies, Vancouver, BC, Canada). The medium was replaced every 48 h until cells reached 70–80% confluence, after which cells were expanded for up to four passages. Human nasal epithelial progenitor cells (hNEpCs) were isolated as previously described by Lim et al. [21].

For organoid formation, hNEpCs were suspended at a density of $1\times 10^{4}$ cells per Matrigel dome in 60% ice-cold Matrigel (growth factor–reduced basement membrane matrix; Corning, Corning, NY, USA) and seeded into 48-well non-treated plates (SPL Life Science, Pocheon, Korea). The Matrigel droplets were polymerized at 37 °C in 5% ${\mathrm{CO}}_{2}$ for 30 min. Airway organoids were subsequently cultured using the PneumaCult^TM^ Airway Organoid Kit (STEMCELL Technologies) according to the manufacturer’s instructions. In this study, Matrigel was used as a standard, non-engineered basement membrane matrix, reflecting the widely adopted practice for airway and mucosal organoid cultures rather than a customized biomaterial formulation.

Cells were maintained in seeding medium for the initial 7 days and then placed into differentiation medium for an additional 28 days. The culture medium was refreshed every other day throughout the culture period. After differentiation, mature airway organoids were treated with house dust mite (HDM) extract (100 μg/mL; Greer Laboratories, Lenoir, NC, USA) for 12 h. Following stimulation, organoids were observed under a bright-field optical microscope to assess morphological changes. Subsequently, organoids were harvested for total RNA extraction and real-time PCR analysis.

### 2.2. RNA Extraction and Quantitative Real-Time PCR

Airway organoids embedded in Matrigel domes were diluted with ice-cold PBS to remove residual Matrigel. During each wash, organoids were carefully pipetted to minimize mechanical damage while ensuring effective Matrigel removal. Total RNA was then extracted using TRIzol reagent (Invitrogen, Carlsbad, CA, USA). For cDNA synthesis, 0.5 μg of total RNA was reverse-transcribed using the iScript^TM^ cDNA Synthesis Kit (Bio-Rad, Hercules, CA, USA).

Quantitative real-time PCR (qPCR) was performed to evaluate the expression of cytokine-related genes using the CFX96^TM^ Real-Time PCR Detection System (Bio-Rad). Reactions were conducted in a total volume of 25 μL containing 2 μL of diluted cDNA, 10 pmol of each primer, and 12.5 μL of iQ^TM^ SYBR Green Supermix (Bio-Rad). Gene expression levels were normalized to glyceraldehyde-3-phosphate dehydrogenase (GAPDH). For downstream classification, qPCR responses were expressed relative to untreated controls, such that a value of 1 indicated the control-equivalent level. In the present study, these dome-level responses were not additionally normalized to dome size, organoid number, or total cell content.

The primer sequences used were as follows: human TSLP (forward, 5′-ATGTTCGCCATGAAAA-3′; reverse, 5′-GCGACGCCACAATCCTTGTA-3′), human IL-33 (forward, 5′-GACTCCTCCGAAACAC-3′; reverse, 5′-CCCAGCTTGAAACACAAGGC-3′), and human GAPDH (forward, 5′-GGCATGGACTGTGGTCATGA-3′; reverse, 5′-TGCACCACCAACTGCTTAGC-3′).

### 2.3. Immunofluorescence Staining

Airway organoids were fixed in 2% (*w*/*v*) paraformaldehyde at room temperature for 30 min, followed by three washes with phosphate-buffered saline (PBS). The fixed organoids were then processed for paraffin embedding. Paraffin-embedded sections (5 μm thick) were deparaffinized in xylene and rehydrated through a graded ethanol series.

For antigen retrieval, sections were immersed in 10 mM citrate buffer (pH 6.0; Sigma-Aldrich, St. Louis, MO, USA) and heated at 95–100 °C for 20 min. After cooling to room temperature, sections were washed with PBS and incubated in 10% normal goat serum (Vector Laboratories, Burlingame, CA, USA) for 1 h to block non-specific binding.

Sections were then incubated overnight at 4 °C with primary antibodies against acetylated α-tubulin (1:400; Santa Cruz Biotechnology Inc., Dallas, TX, USA), MUC5AC (1:400; Abcam, Cambridge, UK), E-cadherin (1:300; Thermo Fisher Scientific, Waltham, MA, USA), p63 (1:400; Abcam), or cytokeratin 5 (1:250; Abcam). The following day, sections were washed and incubated for 1 h at room temperature with species-appropriate secondary antibodies conjugated to Alexa Fluor 488 (green) or Alexa Fluor 546 (red) (1:1000; Molecular Probes, Eugene, OR, USA).

All nuclei were counterstained with 4’,6-diamidino-2-phenylindole (DAPI; Sigma-Aldrich). Fluorescence images were acquired using a confocal laser scanning microscope (LSM 800 with Airyscan; Carl Zeiss Meditec AG, Jena, Germany).

### 2.4. Image Acquisition

Bright-field images of differentiated airway organoids were acquired using an inverted microscope (Nikon Eclipse TS100, Nikon Corporation, Tokyo, Japan) at 40× magnification, equipped with a 6.3-megapixel CMOS sensor camera (JLHC-6M, Nikon Corporation 1/1.8” sensor). To encompass the entire dome area, six overlapping fields of view were captured in a 2 × 3 grid pattern by manually translating the microscope stage. For each sub-region, 2–4 Z-stack images were captured at different focal planes, thus compensating for the three-dimensional structure and uneven thickness of the Matrigel dome.

### 2.5. Statistical Analysis

Quantitative analysis of qPCR data was performed using the $2^{-\Delta \Delta C_{t}}$ method. Each of the data clusters shown in Figure 4A represents a single Matrigel dome without technical or biological replication; therefore, no statistical testing was applied to compare expression levels between domes. For Figure 4B, Pearson’s correlation coefficient (*r*) and the corresponding *p*-value were calculated to assess the relationship between TSLP and IL-33 expression levels.

### 2.6. Segmentation and ROI Definition

**Figure 1:**
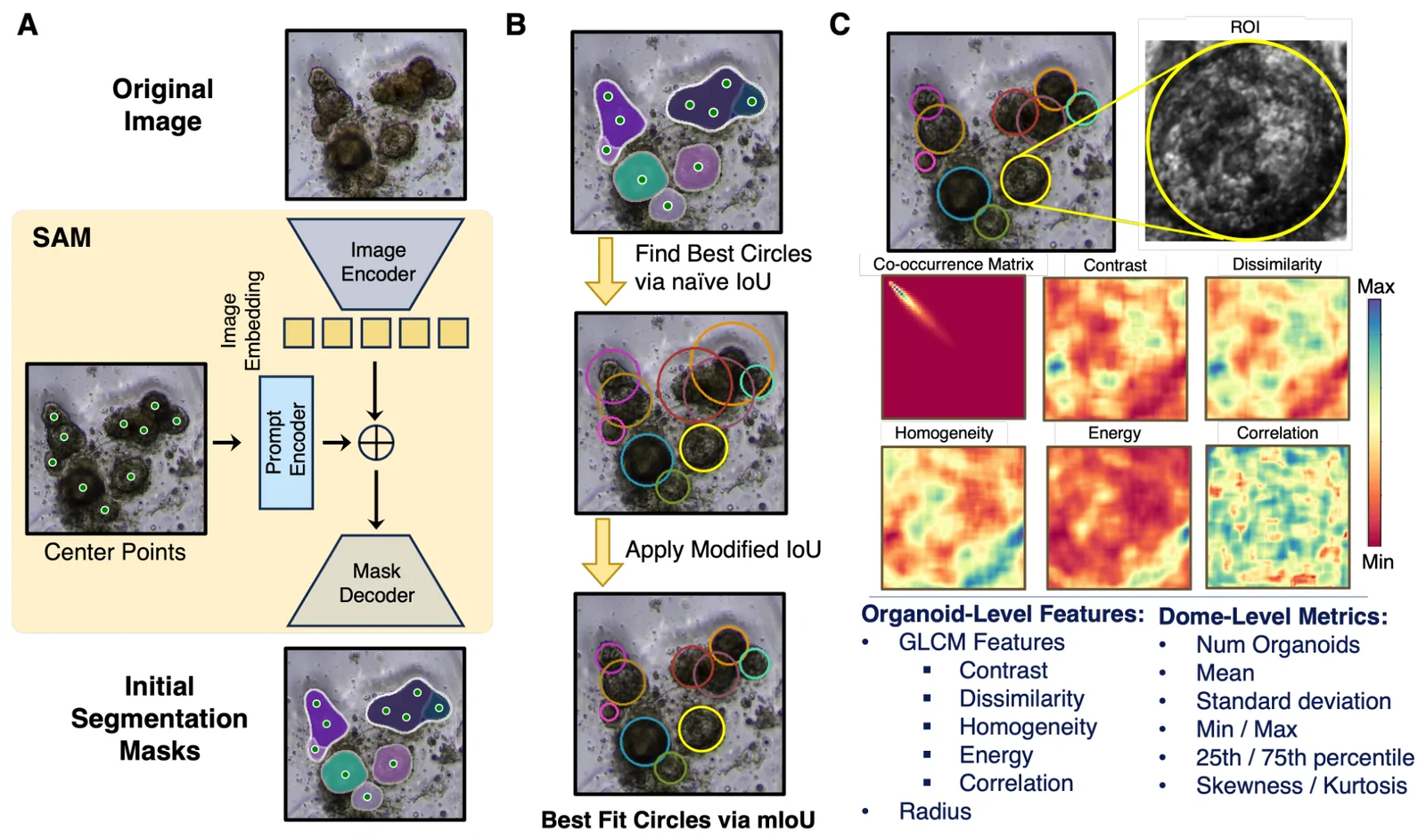
Point prompt-based segmentation and texture-based feature extraction pipeline for airway organoids. (**A**) Initial organoid segmentation using the Segment Anything Model (SAM). Manually annotated center points (green dots) are provided as prompts, producing segmentation masks, with different colors distinguishing individual organoids. (**B**) Geometry-based refinement of individual organoid regions. Colored circles indicate individual organoid ROIs. Naive IoU-based fitting (middle) may cause overestimation or boundary leakage, whereas the proposed penalty-based modified IoU (mIoU) (bottom) yields robust, non-overlapping circular ROIs. (**C**) Texture feature extraction from refined ROIs. The yellow circle indicates a representative ROI used to illustrate texture analysis. GLCM-derived features are extracted from each ROI and aggregated at the dome level using statistical descriptors and organoid count for downstream analysis.

Segmentation of organoids was initially performed using the Segment Anything Model (SAM) [22], with manually annotated center points provided as prompts to the prompt encoder (Figure 1A). This point-prompt strategy allowed SAM to generate initial segmentation masks for individual organoids within each dome. One center-point prompt was placed per organoid, yielding 15,072 prompts across all analyzed dome images (mean 193.2 per image; median 191; range 33–462). All annotations were performed by a single operator, and inter-annotator variability was not formally assessed. Although SAM produced adequate results when organoids were spatially isolated, its outputs became unreliable in crowded regions with multiple organoids in close proximity, often resulting in fragmented masks or merged boundaries (Figure 1A, bottom). Such imperfections hindered the clean delineation of individual organoids required for downstream quantitative analysis. A fully automated segmentation pipeline was considered but not adopted, because densely packed organoids and ambiguous bright-field boundaries frequently reduced segmentation stability under the current imaging conditions.

To address this problem, we imposed a geometry-constrained refinement strategy that leveraged the reliability of the annotated center points and searched for the best-fitting circular region around each center (Figure 1B). For each candidate circle $\chi_{C}\left(R,c\right)$ with radius *R* and center c, the corresponding SAM-derived mask $\chi_{S}$ was treated as a coarse proxy for the underlying organoid region.

The initial approach of circle fitting using the classical intersection-over-union (IoU) metric, which quantifies the overlap between a candidate circle $\chi_{C}$ and the SAM-derived mask $\chi_{S}$ as

(1) $$\mathrm{IoU}\left(\chi_{S},\chi_{C}\right)=\frac{|\chi_{S}\cap \chi_{C}|}{|\chi_{S}\cup \chi_{C}|},$$

provides a measure of spatial overlap, where |·| represents the area of the corresponding region. However, optimizing the circle radius based solely on IoU often resulted in overestimated radii or boundary leakage into adjacent organoids, particularly in densely packed regions in which SAM masks partially overlapped (Figure 1B, middle).

To overcome these limitations, we refined the circle radius by adopting a modified version of IoU, referred to as the modified IoU (mIoU), which incorporates additional penalties for size inconsistencies and excess coverage. The mIoU objective is defined as

(2) $$\mathrm{mIoU}=\mathrm{IoU}\left(\chi_{S},\chi_{C}\right)-\alpha \underset{\mathrm{size}\;\mathrm{mismatch}\;\mathrm{penalty}}{\underset{︸}{\left(\frac{\left|\;\right|\chi_{S}\cup \chi_{C}\left|-\chi_{S}\right|\;|}{|\chi_{S}|}\right)}}-\beta \underset{\mathrm{excess}\;\mathrm{coverage}\;\mathrm{penalty}}{\underset{︸}{\left(\frac{|\chi_{C}\setminus \chi_{S}|}{|\chi_{S}|}\right)}}.$$

This refined approach ensures a more accurate radius estimation by penalizing misalignments and reducing leakage into neighboring regions.

Here, the first term corresponds to the classical IoU, which measures the overlap quality between the candidate circle and the SAM-derived mask. The second term, denoted as the size mismatch penalty, increases when the area of the circle differs substantially from the reference region, thereby discouraging solutions in which the circle is disproportionately large or small. The third term, expressed as the excess coverage penalty, penalizes circle areas that extend beyond the SAM mask, preventing leakage into neighboring organoids or background regions. In practice, the penalty weights were set to α=1.0 and β=0.05, providing a balance between enforcing strict size fidelity and allowing minor tolerance for boundary variations.

The optimization problem was thus formulated as

(3) $$R^{*}=\underset{R\in [R_{\min},R_{\max}]}{\arg\max}\mathrm{mIoU}\;\left(\chi_{S},\chi_{C}\left(R,c\right)\right),$$

which was solved using Powell’s derivative-free method, which is well suited for non-smooth objective functions. This refinement procedure integrated overlap quality with explicit penalties for overestimation and leakage, enabling robust recovery of individual organoids from imperfect SAM outputs. The resulting circle-based ROIs provided compact, geometrically faithful representations of organoids, forming a reliable basis for subsequent feature extraction.

### 2.7. Feature Extraction: Texture Features and Dome-Level Aggregation

Texture information was quantified on a per-organoid basis using gray-level co-occurrence matrices (GLCMs) [23]. For each circle-based ROI, the largest inscribed square region was extracted and converted to grayscale. Co-occurrence matrices were then computed with unit pixel offset at four canonical orientations (0°,45°,90°,135°), normalized to probability distributions, and averaged across orientations to obtain orientation-independent measures. From these GLCMs, five widely used Haralick descriptors [23] were derived: contrast, energy, homogeneity, correlation, and dissimilarity. In addition to these matrix-derived statistics, the geometric radius of each ROI was retained as a shape-related variable, and the total number of organoids detected within a dome was recorded as a morphological descriptor. Thus, every organoid contributed a feature vector characterizing its intrinsic texture and scale.

Because each dome typically contained multiple organoids, dome-level representations required aggregating organoid-level measurements into higher-order statistical descriptors. For every dome, the set of feature values ${\left\{f_{k}^{\left(d\right)}\right\}}_{k=1}^{n_{d}}$ from $n_{d}$ organoids was summarized using a range of descriptive statistics designed to capture both central tendency and distributional variability. Specifically, we computed the mean and standard deviation to reflect the average behavior and dispersion of organoid textures, the minimum and maximum to capture the range of variability, the 25th and 75th percentiles to describe interquartile spread, and higher-order moments such as skewness and kurtosis to account for asymmetry and heavy-tailed distributions. This multi-level aggregation ensured that domes with heterogeneous organoid populations were faithfully represented in the final feature space, rather than being reduced to simple averages.

As a result, a comprehensive dome-level feature vector was achieved, integrating detailed texture information across all organoids within a sample. In total, six features (five GLCM-derived features and radius) were combined with eight statistical descriptors, together with organoid count, yielding 49 dome-level features. These features were then used as inputs for classification against qPCR labels. The full set of features and their aggregation formulas are summarized in Table 1.

### 2.8. Feature Assembly and Model Training

All organoid-level texture descriptors derived from GLCM analysis were aggregated to the dome level, yielding a single feature vector per dome. As summarized in Table 1, each GLCM-derived descriptor (contrast, energy, homogeneity, correlation, dissimilarity, and radius) was combined with statistical features (mean, standard deviation, minimum, maximum, percentiles, skewness, and kurtosis), together with the number of organoids per dome. This procedure produced a structured tabular dataset in which each row represented one dome and each column corresponded to a dome-level statistical descriptor. A total of 78 Matrigel domes derived from two independent donors were included in the machine-learning analysis. Each Matrigel dome was treated as an independent sample, and the corresponding bright-field image features were paired with the qPCR-derived cytokine response labels for supervised model training and evaluation. For supervised learning, cytokine responsiveness was binarized using a predefined threshold of 1.5-fold change relative to untreated controls. Under this criterion, 45 domes were classified as TSLP-positive and 33 as TSLP-negative, whereas 37 domes were classified as IL-33-positive and 41 as IL-33-negative (Appendix A Table A1). This cutoff was selected as a pragmatic threshold to identify domes with meaningful cytokine induction above baseline while maintaining a workable class balance in the present dataset; the effect of neighboring thresholds is summarized in Appendix A Table A3. The resulting feature table was aligned with the qPCR labels for TSLP and IL-33, enabling supervised learning at the dome level.

**Figure 2:**
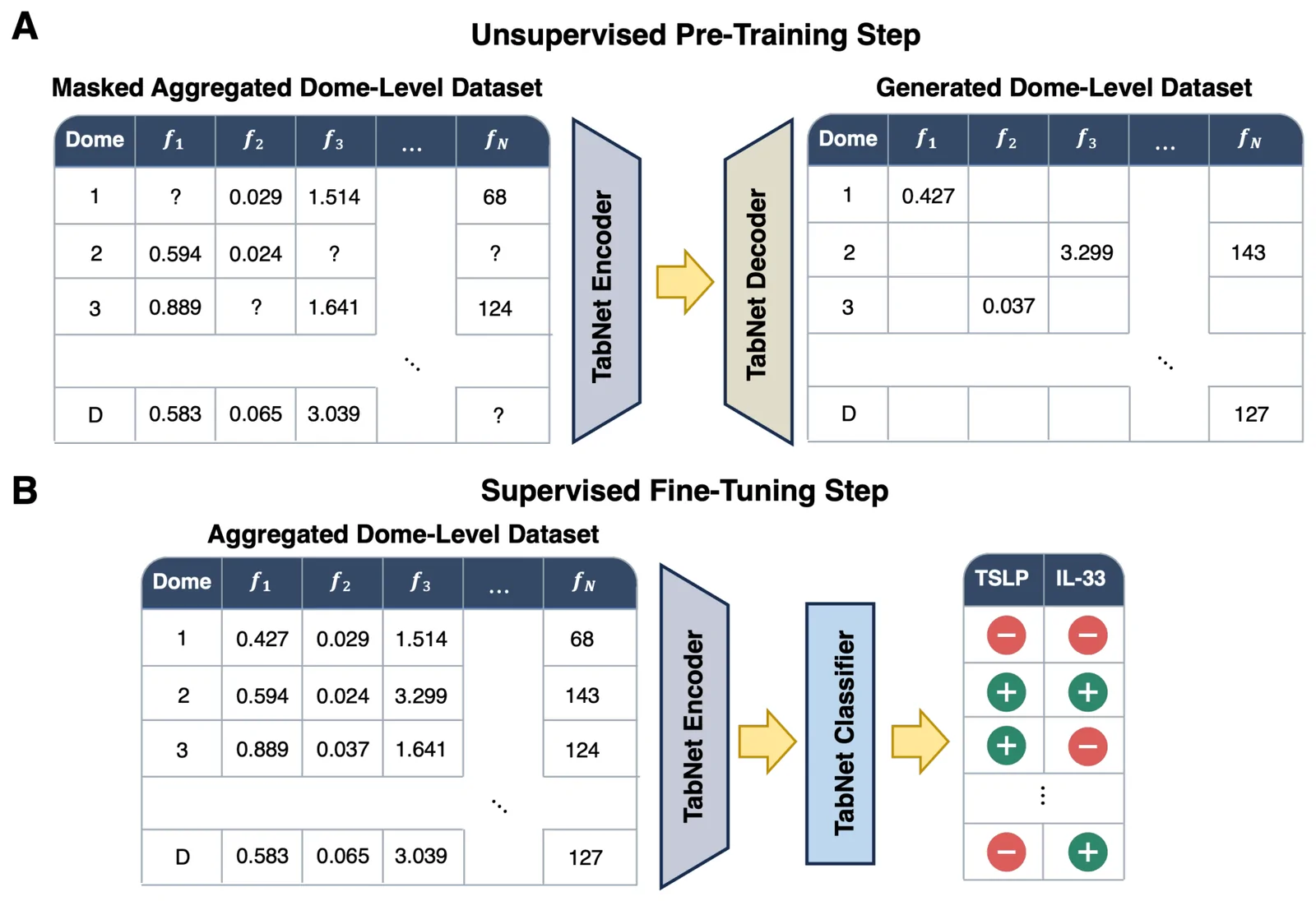
Schematic illustration of the dome-level learning framework based on TabNet. (**A**) Tabular representation of aggregated dome-level features and unsupervised feature transformation through a TabNet encoder, where “?” denotes masked feature values during pre-training. (**B**) Supervised classification of dome-level inflammatory responsiveness using a TabNet classifier, yielding binary predictions for TSLP and IL-33, where “+” and “−” denote positive and negative cytokine responses, respectively.

To exploit this structured representation, we employed TabNet, a deep tabular learning architecture that integrates sequential attention masks to learn feature selection and nonlinear interactions. TabNet was used in two stages: first, the network performed a feature transformation step (Figure 2A) to learn compact latent embeddings of dome-level descriptors; second, these embeddings were passed to a classification head to discriminate between positive and negative domes for each cytokine marker (Figure 2B). Model performance was evaluated using stratified 8-fold cross-validation at the dome level. In each iteration, the dataset was randomly divided into eight stratified folds while preserving the class distribution. Seven folds were used for model training and one fold for validation. Data preprocessing, including feature transformation and standardization, was performed using only the training data within each fold and subsequently applied to the corresponding validation data to prevent information leakage. Model performance was evaluated by averaging the results across all eight validation folds. Because the present dataset contained only two independent donors, donor-stratified or leave-one-donor-out validation was not used as the primary evaluation strategy; the reported metrics therefore reflect dome-level performance within the current dataset. The hyperparameter configuration used for TabNet was specified before the main cross-validation analysis and retained across all folds. Baseline models were evaluated under the same cross-validation and preprocessing framework using fixed model settings (Appendix A Table A4). Although the maximum training length was set to 2000 epochs, training was controlled by early stopping with a patience of 150 epochs. In addition, TabNet employed sparse feature masking with sparsity regularization ($\lambda_{\mathrm{sparse}}=2\times 10^{-3}$), and class-balanced sample weighting was applied during optimization to improve generalization under the limited-data setting.

## 3. Results

### 3.1. Respiratory Organoid Development and Characterization in Matrigel-Based 3D Culture

**Figure 3:**
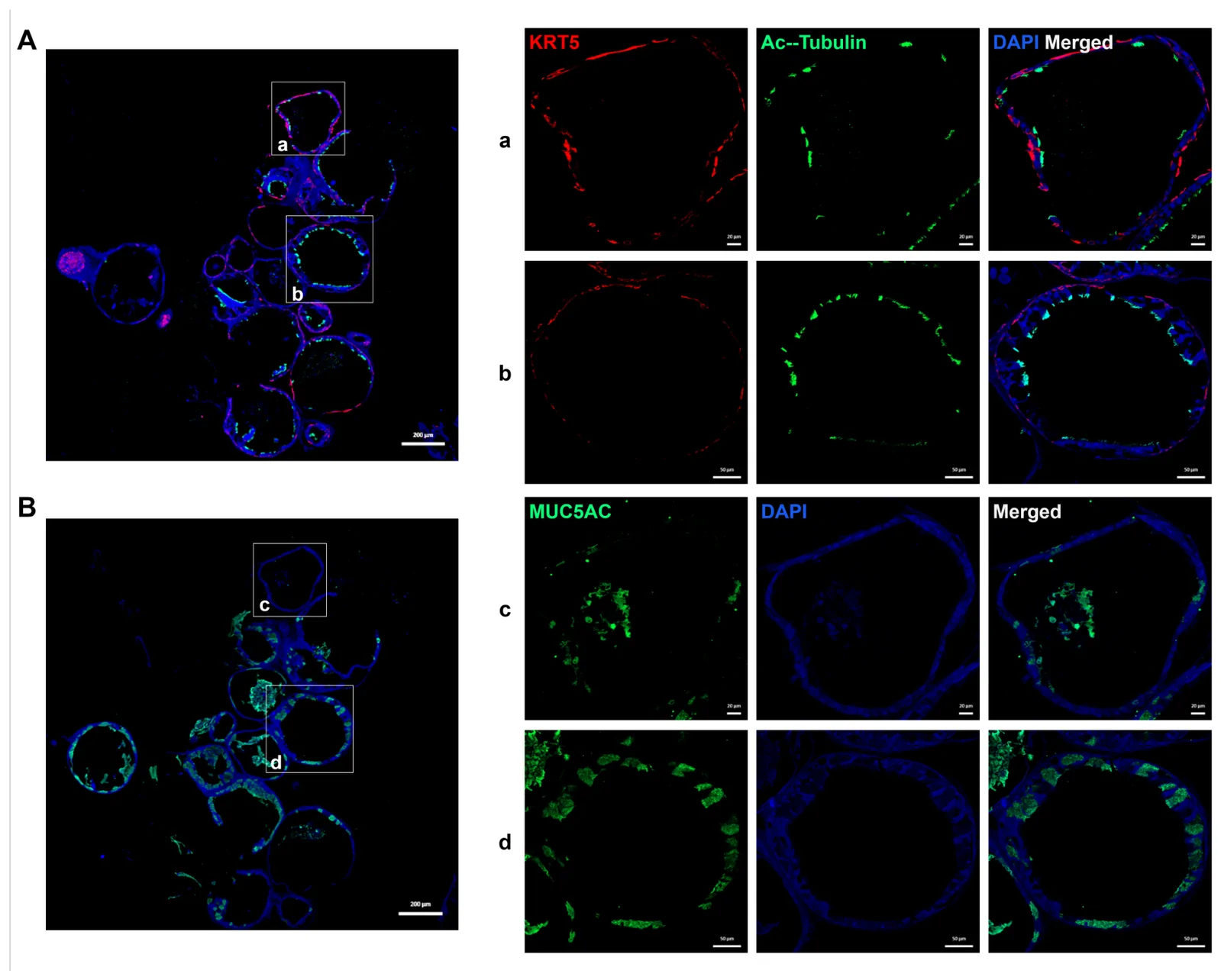
Immunofluorescence staining of airway organoids cultured under Matrigel-based three-dimensional conditions. (**A**) Representative image showing co-staining of the ciliated cell marker acetylated α-tubulin (green) and the basal cell marker KRT5 (red). (**B**) Immunostaining of the goblet cell marker MUC5AC (green). Nuclei were counterstained with DAPI (blue). Organoids exhibit a spherical morphology and show variable expression of epithelial lineage markers within the same Matrigel domes. Boxed regions are shown at higher magnification in panels (**a**–**d**). ROI-based fluorescence analysis confirming marker variability is provided in Appendix A Figure A1. Scale bars: 200 μm (overview images in (**A**,**B**)); 20 μm (panels (**a**,**c**)); 50 μm (panels (**b**,**d**)).

To evaluate the structural and cellular characteristics of airway organoids cultured in a Matrigel-based 3D system, immunofluorescence staining was performed using lineage-specific markers. The organoids exhibited a spherical morphology and developed a pseudostratified epithelial structure within the Matrigel domes. Immunostaining revealed the presence of key airway epithelial cell markers, including the goblet cell marker MUC5AC, the ciliated cell marker acetylated α-tubulin, and the basal cell marker keratin-5 (KRT5) (Figure 3). These markers were spatially localized in a manner consistent with the organization of native human airway epithelium. The detection of these specific cell types confirmed that the airway organoids recapitulate essential cellular components of the respiratory tract and demonstrate successful differentiation in the 3D Matrigel culture system.

**Figure A1:**
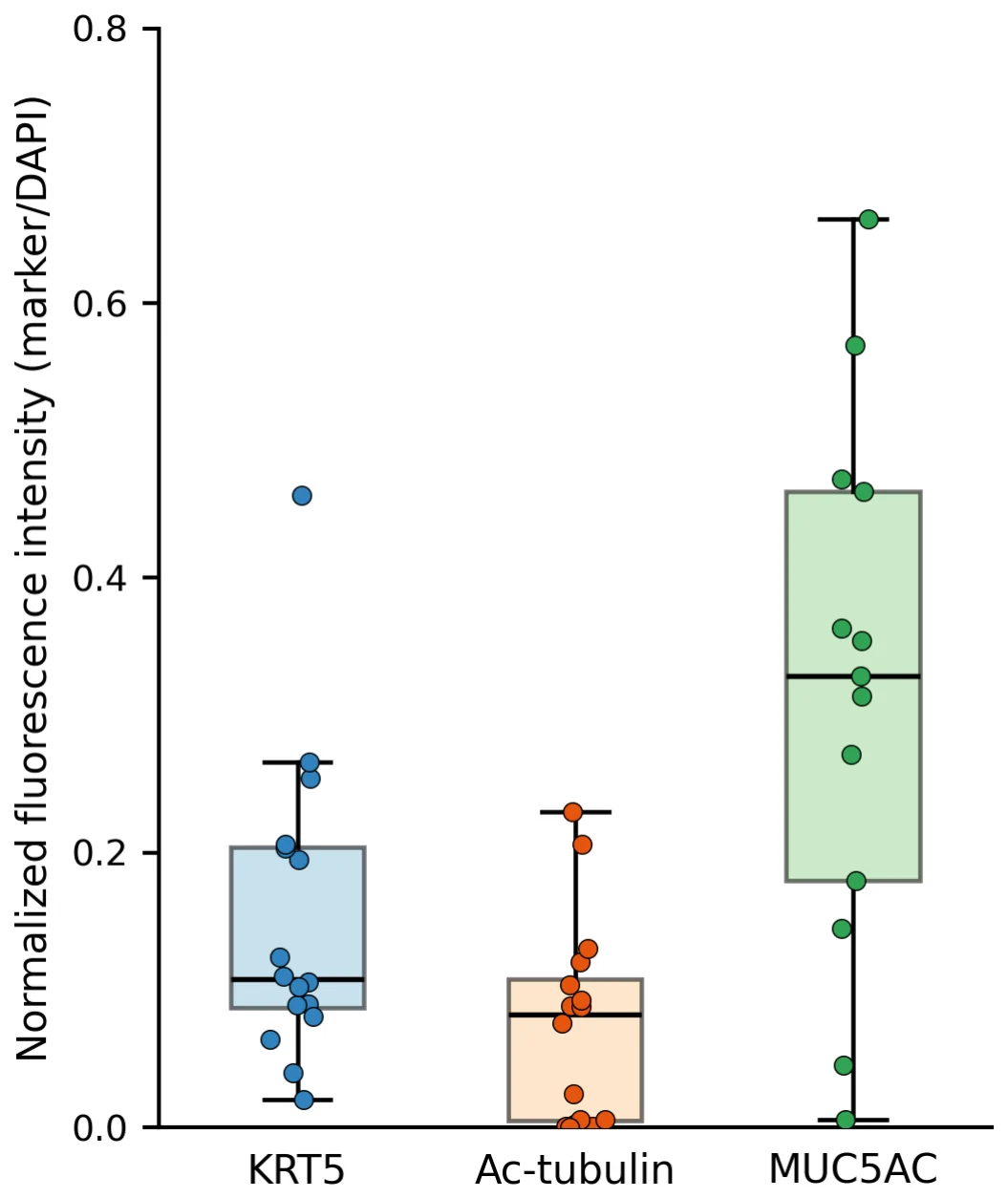
Quantitative assessment of differentiation marker variability across airway organoids. ROI-based ImageJ (version 1.53t) quantification of normalized fluorescence intensity for KRT5, acetylated α-tubulin, and MUC5AC across individual organoids. Each point represents one organoid.

However, despite the presence of lineage-specific cell types, the degree of epithelial differentiation varied among organoids within individual Matrigel domes. Notably, distinct marker expression profiles were observed between organoids cultured within the same dome. As shown in Figure 3, some organoids exhibited strong expression of ciliated or goblet cell markers, whereas others in the same dome displayed weak or undetectable staining. These findings highlight substantial heterogeneity in epithelial maturation among organoids sharing the same culture conditions. Quantitative ROI-based Fluorescence analysis further supported substantial variability in differentiation marker expression across organoids (Appendix A Figure A1 and Appendix A Table A2). Such variability between organoids within a single dome is likely to contribute to dome-to-dome variability in functional responses, thereby potentially compromising the accuracy and reproducibility of experimental outcomes. To mitigate this limitation, we implemented a selection strategy based on organoid responses to HDM extract, enabling the identification of Matrigel domes containing organoids that exhibited consistent and robust inflammatory responses.

### 3.2. HDM Stimulation Induces Pro-Inflammatory Cytokine Expression in Airway Organoids

**Figure 4:**
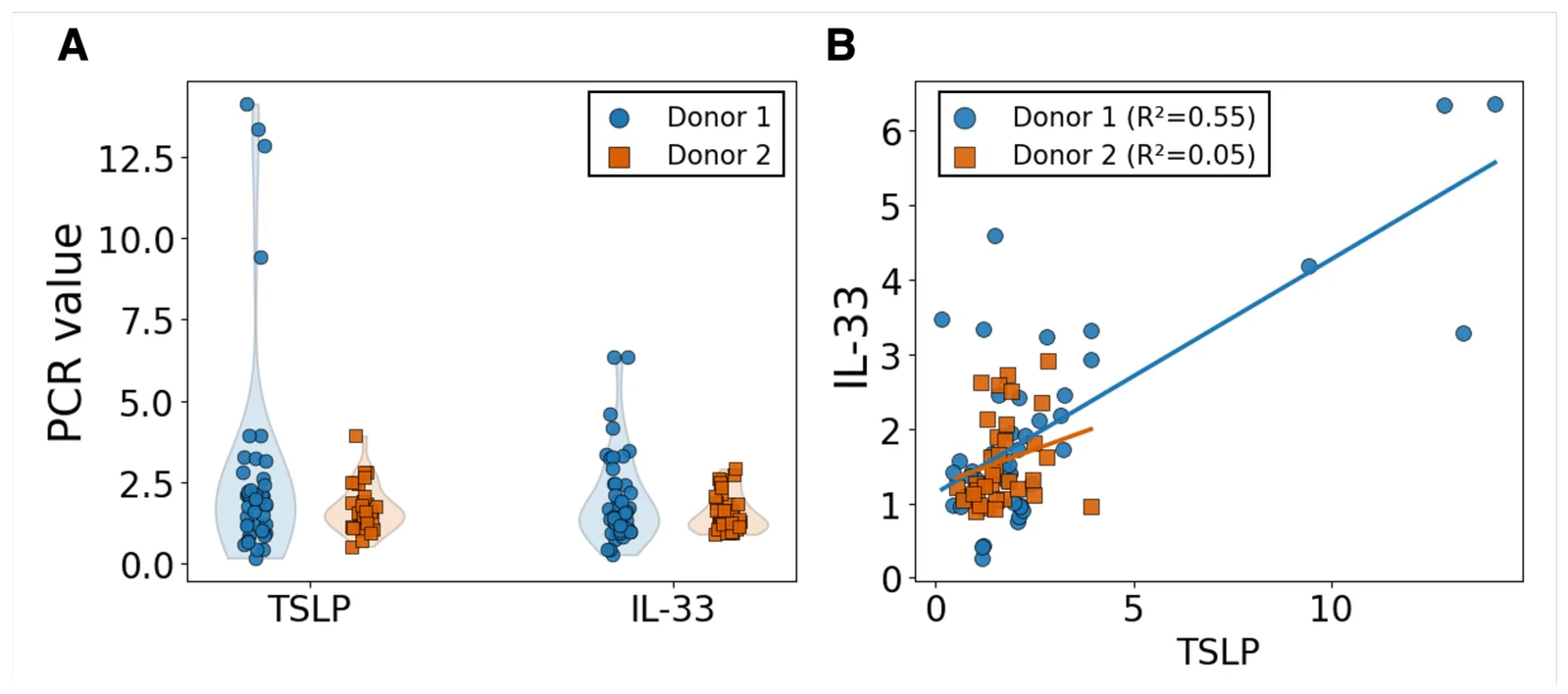
Heterogeneity and donor-specific distribution of cytokine responses across airway organoid domes following HDM stimulation. (**A**) qPCR-measured expression levels of TSLP and IL-33 at the individual dome level 12 h after HDM stimulation, shown as violin plots with overlaid donor-specific data points. Each point represents one Matrigel dome, and donor identity is indicated by color and marker shape, and the shaded areas represent the distribution of expression values. (**B**) Relationship between TSLP and IL-33 expression across domes, shown separately for each donor. The blue and orange solid lines represent the donor-specific linear regression fits for Donor 1 and Donor 2, respectively, with the corresponding $R^{2}$ values shown in the legend.

To evaluate the inflammatory response of airway organoids, Matrigel domes were stimulated with HDM extract for 12 h. The mRNA expression levels of thymic stromal lymphopoietin and interleukin-33 (IL-33) were quantified at the individual dome level using the $2^{-\Delta \Delta C_{t}}$ method and are shown as violin plots to assess dome-to-dome variability (Figure 4A).

Both cytokines exhibited a wide range of expression levels across domes, indicating substantial heterogeneity in the inflammatory response to HDM stimulation. Although the majority of domes showed modest induction, a subset displayed markedly elevated expression of either TSLP or IL-33, suggesting intrinsic variability in inflammatory potential within the same culture conditions. Notably, the distribution of TSLP expression was broader than that of IL-33, indicating greater variability in epithelial-derived TSLP induction.

Correlation analysis revealed a significant positive association between TSLP and IL-33 expression across individual domes (Figure 4B; Pearson r=0.661, *p* < 0.0001), suggesting partial coordination in cytokine regulation at the dome level. By integrating these gene expression profiles with bright-field images analyzed using a deep learning-based approach, we further identified Matrigel domes containing organoids that exhibited consistent inflammatory responses to HDM stimulation.

### 3.3. Deep Learning–Based Classification of Inflammatory Responsiveness

We used TabNet [24], a deep learning architecture designed for tabular data (obtained using quantitative morphological and textural features extracted from bright-field images of airway organoid domes), as the primary classification model. Because qPCR was measured from the pooled response of all organoids within each dome, both the prediction target and the image-derived features were defined at the dome level. The model was trained to classify individual domes based on their positive or negative qPCR inflammatory responses. Domes were classified as positive or negative for each cytokine target using a predefined 1.5-fold threshold relative to untreated controls, and model performance was evaluated using stratified 8-fold cross-validation at the dome level. The proposed framework demonstrated consistently strong performance across both prediction tasks.

**Figure 5:**
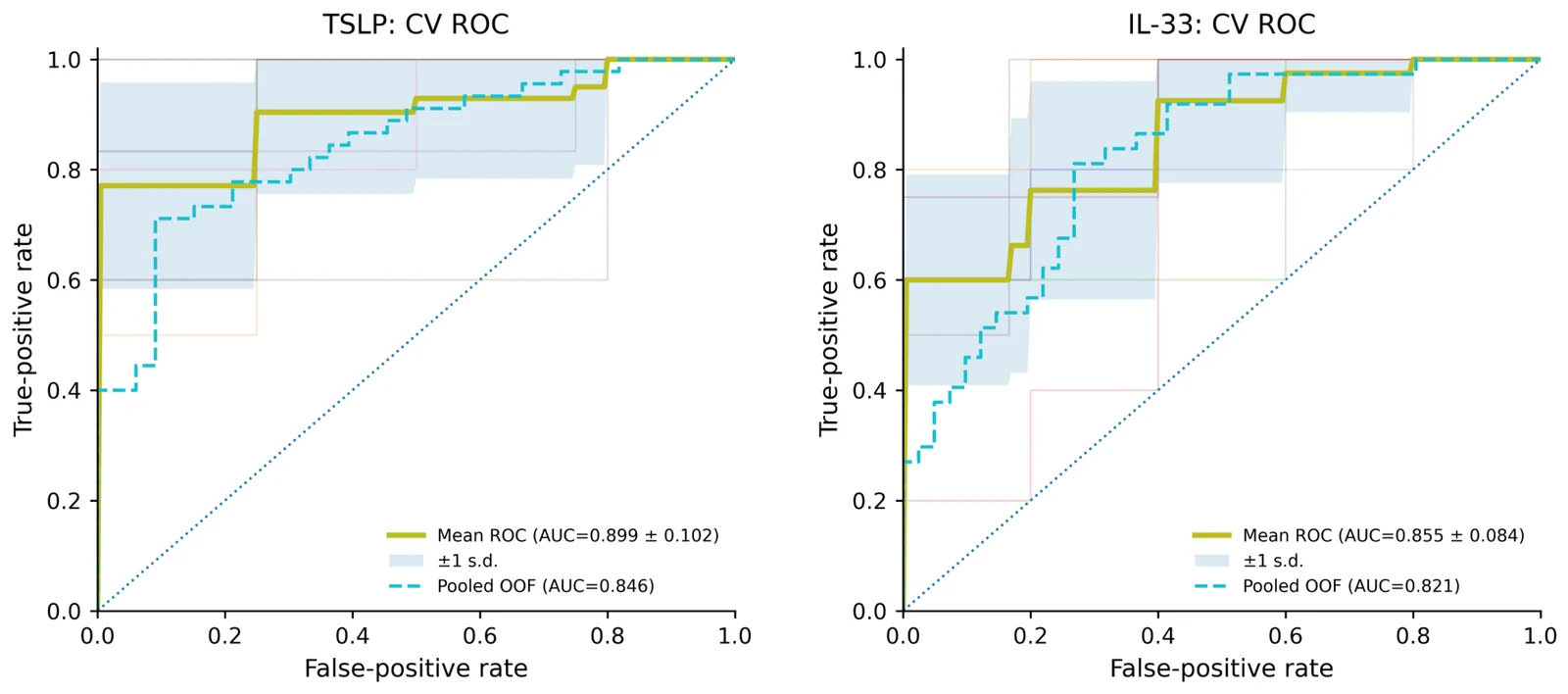
Averaged ROC curves across eight stratified cross-validation folds for predicting TSLP (**left**) and IL-33 (**right**) responses. The yellow-green solid line represents the mean ROC curve, the light-blue shaded region indicates ±1 standard deviation across folds, the cyan dashed line represents the pooled out-of-fold ROC curve, and the blue dotted diagonal line indicates chance-level performance.

**Figure 6:**
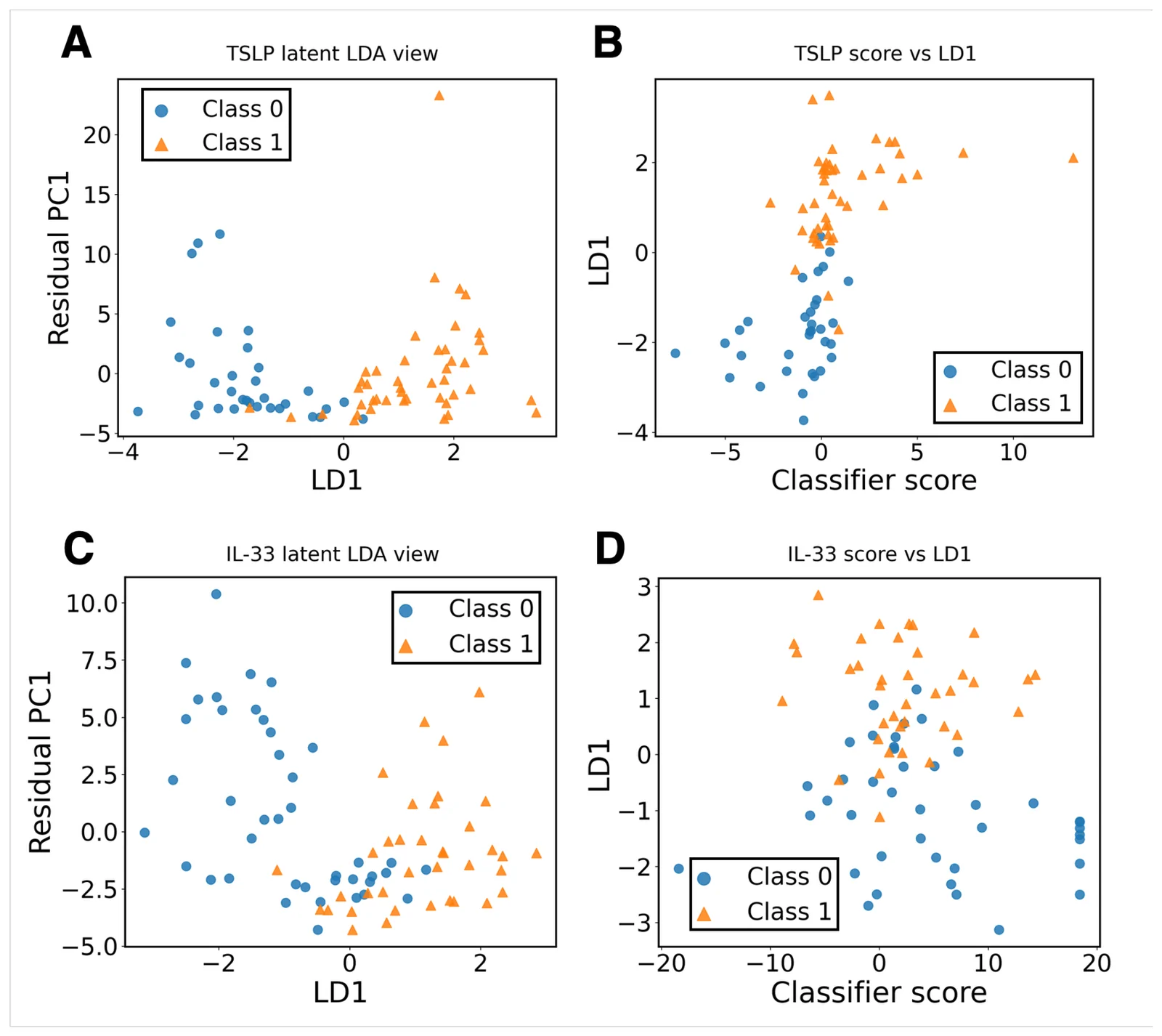
Low-dimensional visualization of dome-level feature structure associated with qPCR-defined inflammatory responsiveness. (**A**,**B**) TSLP and (**C**,**D**) IL-33. (**A**,**C**) show projection of dome-level image descriptors onto the primary linear discriminant axis (LD1) and the leading residual component (Residual PC1), whereas (**B**,**D**) show the relationship between classifier score and LD1. Class 0 and Class 1 indicate the negative and positive qPCR response groups, respectively.

For TSLP, it achieved the highest balanced accuracy (0.910 ± 0.066) and F1-score (0.911 ± 0.078), indicating reliable discrimination between positive and negative domes despite class imbalance. For IL-33, the model achieved a balanced accuracy of 0.833 ± 0.048 with similarly stable performance across cross-validation folds. Given that all domes originated from only two donors, these results should be interpreted as demonstrating consistent dome-level classification performance under the present cross-validation setting rather than donor-independent generalization. To provide a more representative assessment of model performance, Figure 5 presents the mean ROC curves obtained across the eight stratified cross-validation folds, with the shaded regions representing ±1 standard deviation. Table 2 summarizes the fold-wise performance as mean ± standard deviation and additionally reports the corresponding 95% confidence intervals for ROC-AUC, balanced accuracy, F1-score, and accuracy. To further examine the class-related structure of the dome-level descriptors, we projected the feature space using linear discriminant analysis and residual variation decomposition (Figure 6). The two response groups showed clear separation along the primary discriminant axis while retaining within-class heterogeneity, and the classifier score was positively aligned with this discriminant direction, indicating that the extracted features captured biologically relevant variation associated with cytokine responsiveness. These results collectively show that our model effectively captures structural and textural cues linked to cytokine expression, enabling accurate classification of qPCR responsiveness.

To interpret the TabNet prediction mechanism, dome-level classification can be viewed as a two-stage mapping in which organoid-level descriptors are first aggregated into a dome-level representation and subsequently classified by TabNet. Formally, the aggregation operator A(·) summarizes the organoid-level descriptor values of dome *d* using their mean, standard deviation, minimum, maximum, 25th and 75th percentiles, skewness, and kurtosis, together with the organoid count,

(4) $$\Phi^{\left(d\right)}=A\left({\left\{f_{k}^{\left(d\right)}\right\}}_{k=1}^{n_{d}}\right)\in R^{49},$$

yielding a feature vector that jointly encodes the representative morphological and textural properties of dome *d* together with the heterogeneity among its constituent organoids. The inflammatory responsiveness of dome *d* is then predicted as

(5) $${\hat{y}}^{\left(d\right)}=g_{\theta }\left(\Phi^{\left(d\right)}\right),$$

where $g_{\theta }$ denotes the trained TabNet classifier, which performs the prediction through a sequence of decision steps in which dome-specific attention masks selectively emphasize informative subsets of the descriptors,

(6) $$g_{\theta }\left(\Phi^{\left(d\right)}\right)=\sum_{t=1}^{T}h_{t}\left[M_{t}\left(\Phi^{\left(d\right)}\right)⊙\Phi^{\left(d\right)}\right],$$

where $M_{t}\left(\Phi^{\left(d\right)}\right)\in {\left[0,1\right]}^{49}$ denotes the attention mask generated at decision step *t*, ⊙ denotes the element-wise product, and $h_{t}\left(\;\cdot \;\right)$ denotes the nonlinear feature transformation applied to the masked representation. Because this mask is a function of the input feature vector itself, it can vary across domes, allowing different morphological and textural descriptors to contribute to the prediction of different domes rather than relying on a single fixed feature weighting. This adaptive feature-selection mechanism is particularly suitable for heterogeneous airway organoid datasets, where the descriptors most relevant to cytokine responsiveness may vary across individual Matrigel domes. To ensure leakage-free evaluation, all preprocessing operations were estimated exclusively from the training partition of each cross-validation fold and subsequently applied to the corresponding validation partition,

(7) $${\tilde{\Phi }}^{\left(d,s\right)}=T_{s}\left(\Phi^{\left(d\right)}\right),\;T_{s}=T\left(S_{s}^{\mathrm{train}}\right),$$

where $S_{s}^{\mathrm{train}}$ denotes the training subset of fold *s*, ensuring that validation domes never influenced the preprocessing parameters applied to them.

This aggregation–representation–prediction framework, combined with the leakage-free evaluation protocol, is consistent with the low fold-to-fold standard deviation observed across all four metrics in Table 2, supporting the stable dome-level performance of the proposed model.

### 3.4. Attention-Based Feature Importance Analysis

To evaluate the interpretability of TabNet, we performed a feature importance analysis based on the sequential attention masks learned during prediction. In TabNet, the global importance of feature *j* is defined as

(8) $$I_{j}=\sum_{d=1}^{D}\sum_{t=1}^{T}M_{t,j}^{\left(d\right)},$$

where $M_{t,j}^{\left(d\right)}$ denotes the *j*-th component of the attention mask $M_{t}\left(\Phi^{\left(d\right)}\right)$ in Equation (6), i.e., the value assigned to feature *j* for dome *d* at decision step *t*; *T* is the number of decision steps, and *D* is the number of evaluated domes. Feature importance scores were aggregated across cross-validation folds and ranked accordingly.

**Figure 7:**
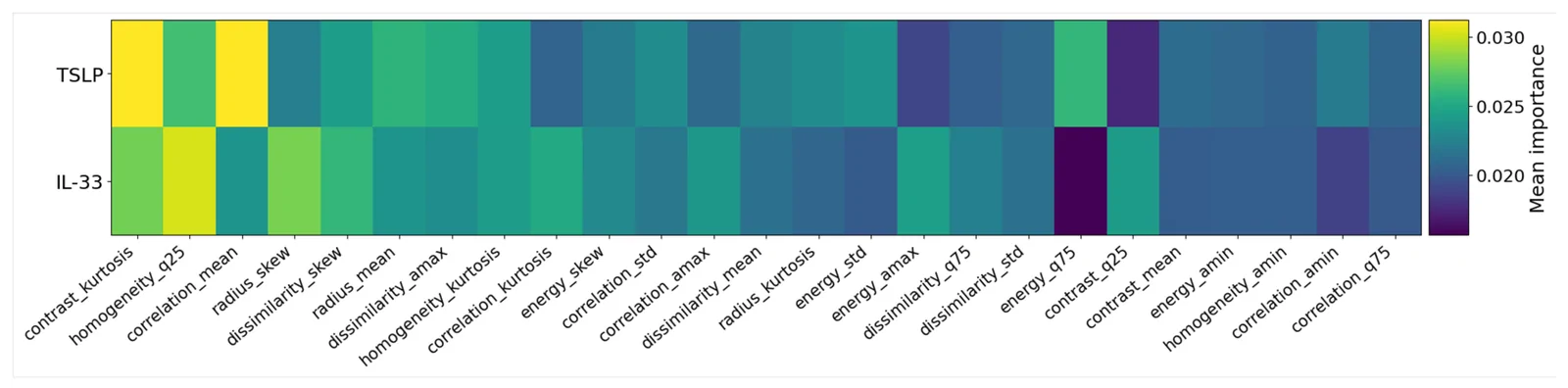
Comparative attention-based feature importance rankings for TSLP and IL-33 predicted by TabNet. The top 25 features are displayed as a horizontal heatmap. Columns represent individual features and rows represent prediction targets. Color intensity reflects the mean attention-based importance score aggregated across cross-validation folds, with brighter values indicating higher importance.

For TSLP prediction, the top-ranked features included contrast_kurtosis, correlation_mean, homogeneity_q25, radius_mean, dissimilarity_skew, and radius_kurtosis. For IL-33, homogeneity_q25, radius_skew, contrast_kurtosis, dissimilarity_skew, correlation_kurtosis, and correlation_mean ranked highest (Figure 7). Notably, several features (e.g., contrast_kurtosis, homogeneity_q25, correlation_mean) were ranked highly for both targets, suggesting that a shared set of morphological characteristics underlies the prediction of both cytokine responses. From a biological perspective, top-ranked texture features such as contrast_kurtosis and homogeneity_q25 suggest that local spatial heterogeneity within organoids, likely reflecting differences in epithelial folding, lumen structure, and cellular packing, is informative for predicting cytokine induction. These morphological characteristics are closely related to epithelial maturation and differentiation state. Geometric descriptors such as radius_mean and radius_kurtosis further indicate that variability in organoid size and shape contributes to dome-level prediction, possibly reflecting differences in growth dynamics or structural development [25,26].

## 4. Discussion

The present study combined a Matrigel-based airway organoid platform with imaging-guided selection to address a fundamental challenge in organoid research—intrinsic heterogeneity in epithelial differentiation and functional responses. Although the organoids successfully recapitulated key structural and cellular features of the native airway epithelium, including the presence of goblet, ciliated, and basal cell lineages, we identified marked variability in lineage marker expression among organoids within the same dome. This intra-dome heterogeneity extended to functional outcomes, as evidenced by the broad and inconsistent induction of TSLP and IL-33 following HDM stimulation. Such variability poses a critical challenge to experimental reproducibility because dome-to-dome differences in inflammatory responsiveness can obscure biologically meaningful effects. Here, we extend image-based organoid analysis beyond descriptive phenotyping by demonstrating its utility for identifying dome-level inflammatory responsiveness and supporting the prospective selection of functionally consistent domes.

IL-33 and TSLP are well-established epithelium-derived cytokines associated with airway allergic inflammation [27,28]. These alarmin cytokines are primarily secreted by epithelial cells and activate dendritic cells and group 2 innate lymphoid cells (ILC2s), thereby promoting Th2-mediated inflammation [29,30]. In addition, numerous in vitro and in vivo studies using HDM extract [31,32,33] have shown that exposure of the airway epithelium to HDM induces the release of epithelium-derived cytokines, which in turn promotes airway hyperresponsiveness and inflammation [32].

The differential responses observed for IL-33 and TSLP may be attributable to distinct activation mechanisms governing their induction in airway epithelial cells. Although both cytokines are classified as epithelium-derived alarmins released in response to cellular damage or external stimuli, the specific triggers and intracellular signaling pathways involved in their regulation differ. In particular, IL-33 and TSLP can be induced by components of HDM extract, including protease-active allergens such as Der p 1 and Der f 1. These proteases can disrupt epithelial barrier integrity or activate protease-activated receptors, thereby promoting the transcriptional upregulation and release of IL-33 and TSLP [34]. These mechanistic differences may partly explain the divergent cytokine expression profiles observed following HDM stimulation in our organoid model.

The heterogeneous induction of IL-33 and TSLP following HDM stimulation likely reflects differences among organoids, even within the same Matrigel dome, in epithelial differentiation status, cellular composition, or functional maturation. Such heterogeneous inflammatory responsiveness represents an important limitation of organoid-based airway inflammation models because organoid-level variability can compromise experimental reproducibility and obscure biologically meaningful effects. In this context, the imaging-guided strategy proposed in this study provides a practical approach for enriching domes with more consistent inflammatory responses, thereby improving the reliability of HDM-induced inflammatory analyses.

By integrating quantitative cytokine measurements with deep-learning analysis of bright-field images, the proposed model achieved strong dome-level classification performance under cross-validation. The results indicate that quantitative morphological and textural descriptors are associated with qPCR-defined cytokine responsiveness. This image-based framework may support the prospective identification of responsive domes without requiring destructive molecular assays before selection. Domes classified as negative represent lower-responsive organoid populations under HDM stimulation and may provide a comparison group for investigating functional heterogeneity. The present framework was restricted to quantitative features extracted from routine bright-field images to evaluate a label-free and non-destructive prediction strategy. Integrating complementary biological measurements may improve both predictive performance and biological interpretation.

To assess the relative performance of the proposed framework, TabNet was compared with conventional machine-learning classifiers, an MLP, and a CNN using the same stratified 8-fold cross-validation protocol. TabNet achieved the highest mean accuracy, balanced accuracy, precision, and F1-score for both cytokine targets, whereas Random Forest showed the highest recall for IL-33 (Table 3). The CNN, which was trained directly on bright-field dome images, also showed lower mean accuracy, balanced accuracy, and F1-score than the GLCM+TabNet framework for both targets. Exploratory paired Wilcoxon signed-rank tests based on fold-wise scores yielded unadjusted two-sided *p*-values below 0.05 for most comparisons between TabNet and the conventional models, except for the TSLP–MLP and IL-33–RF F1-score comparisons (Appendix A Table A5). Because no correction for multiple comparisons was applied, these statistical results should be interpreted cautiously.

Beyond its predictive performance, the feature-based framework enabled interpretation through explicit morphological and textural descriptors. TabNet’s attention-based rankings identified contrast_kurtosis, homogeneity_q25, and radius-related features as important contributors to prediction. These descriptors may capture variations in epithelial organization, lumen structure, cellular packing, and organoid development [25,26]. However, the reliability of these image-derived descriptors depends on accurate organoid detection and ROI definition. Manual center-point prompting was therefore retained because fully automated methods were less reliable for densely packed or touching organoids under the current imaging conditions. The semi-automatic workflow also enabled imaging artifacts and non-organoid structures to be excluded before feature extraction. Nevertheless, this procedure increased the annotation burden and may have introduced operator dependence. Future studies should assess inter-annotator variability and develop automated detection methods that reduce manual effort while preserving reliable ROI definition.

Despite the promising results, several limitations remain. First, because Matrigel is a biologically derived and compositionally variable extracellular matrix, batch-to-batch differences in growth factor composition and mechanical properties can affect organoid growth, differentiation, and morphology. Such variability may influence the image features underlying TabNet predictions and contribute to heterogeneity in dome-level inflammatory responses, thereby limiting the consistency and scalability of the model. Future studies should evaluate defined extracellular matrices or batch-calibration approaches to improve the robustness of morphology–response relationships across different Matrigel lots.

Second, our current organoid system consists exclusively of epithelial cells and therefore does not capture the full complexity of the airway microenvironment, particularly the dynamic interactions among epithelial, immune, and stromal cell populations that shape in vivo inflammatory responses.

Third, the morphology–response relationships identified in this study were derived from a specific organoid culture system. Whether similar relationships apply across disease states, culture conditions, or alternative organoid protocols remains to be determined.

**Figure A2:**
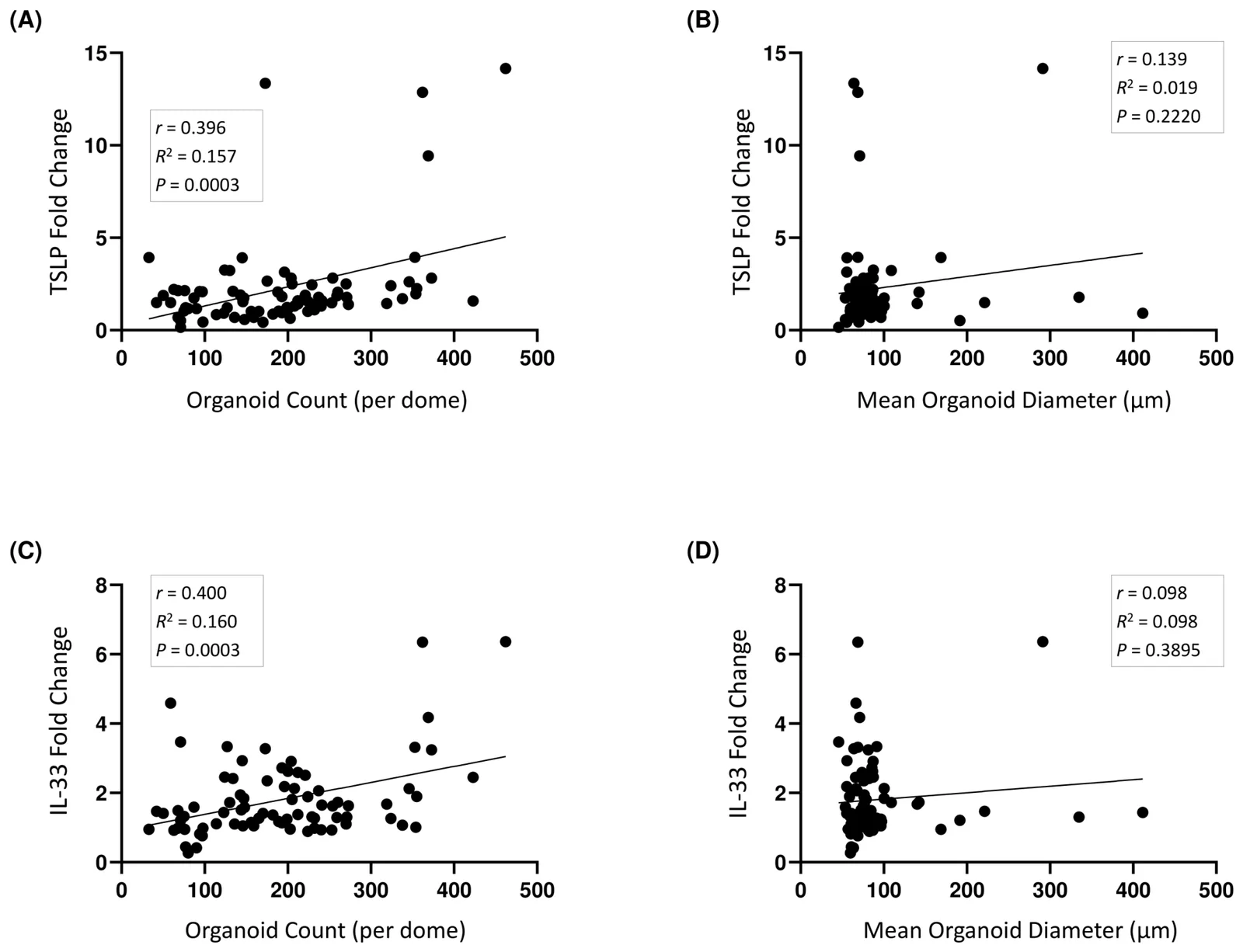
Correlation analysis between dome-level cytokine expression and organoid composition. Pearson correlation analysis was performed between qPCR-derived cytokine expression ($2^{-\Delta \Delta C_{t}}$ values relative to untreated controls) and organoid composition. (**A**) TSLP expression versus organoid count. (**B**) TSLP expression versus mean organoid diameter. (**C**) IL-33 expression versus organoid count. (**D**) IL-33 expression versus mean organoid diameter. Solid lines indicate linear regression fits. Pearson correlation coefficient (*r*), coefficient of determination ($R^{2}$), and corresponding *p*-values are shown in each panel.

Fourth, the present study adopted a predefined 1.5-fold qPCR threshold as an operational criterion for supervised classification rather than as a biologically established definition of inflammatory responsiveness. Because no universally accepted threshold exists for HDM-induced cytokine responses in airway organoids, this cutoff was selected pragmatically before model training to distinguish cytokine induction above baseline while maintaining a workable class balance. Although alternative thresholds influenced the absolute classification performance (Appendix A Table A3), future studies using larger datasets together with independent biological validation will be required to establish biologically meaningful response thresholds. To assess this potential confounding effect, we additionally examined the relationships between cytokine expression and organoid composition. Cytokine expression showed weak positive correlations with organoid count and no significant association with mean organoid diameter (Appendix A Figure A2). These findings suggest that dome composition may have contributed to the measured dome-level responses, although the observed associations were relatively weak. Because RNA was extracted from entire Matrigel domes, the qPCR data should be interpreted as integrated dome-level inflammatory responses rather than per-cell cytokine activity. Future studies incorporating normalization to total protein, DNA content, or direct cell number measurements will further distinguish compositional effects from intrinsic inflammatory responsiveness.

Fifth, the 78 Matrigel domes were obtained from only two donors, and no independent validation cohort was available. Because domes from the same donor could appear in different cross-validation folds, the reported results reflect dome-level discrimination within the present dataset and do not establish donor-independent generalizability. The identified morphology-based signatures should therefore be interpreted as candidate image-derived features associated with qPCR-defined responsiveness under the current experimental conditions rather than as validated biomarkers. Larger multi-donor cohorts, donor-level holdout validation, independent culture batches, nested model-selection procedures, and external datasets will be needed to assess the robustness and generalizability of the proposed framework. Lastly, because the present model was developed exclusively using HDM-stimulated organoids, its applicability to other inflammatory triggers remains uncertain. The predictive features identified here may reflect a combination of general epithelial responsiveness, differentiation status, organoid composition, and HDM-specific morphological signatures. Future studies using additional stimuli, including orthogonal triggers such as viral mimetics of cytokine-driven stimulation, will be required to determine the broader generalizability of this framework.

Despite these limitations, this study takes an important conceptual and methodological leap by integrating airway organoid biology with deep learning-driven image analysis, thereby addressing intrinsic heterogeneity—a major practical obstacle—in organoid-based research. In addition to its relevance for respiratory disease modeling, the imaging-guided dome selection framework may be adaptable to other mucosal organoid systems of the upper aerodigestive tract, where standardized identification of functionally responsive organoids could improve experimental consistency and support future translational applications. By enabling the selection of functionally consistent organoids, our approach enhances the reliability, reproducibility, and translational potential of airway organoid models and lays the groundwork for more precise investigations of respiratory inflammation and disease mechanisms.

## Figures and Tables

**Table 1:** GLCM-based features and dome-level statistical aggregation formulas. P(i,j) denotes the normalized gray-level co-occurrence matrix.

Feature	Formula	Statistical Feature	Formula
Contrast	$\sum_{i=0}^{N_{g}-1}\sum_{j=0}^{N_{g}-1}{\left(i-j\right)}^{2}P\left(i,j\right)$	Mean	${\bar{f}}^{\left(d\right)}=\frac{1}{n_{d}}\sum_{k=1}^{n_{d}}f_{k}^{\left(d\right)}$
Energy	$\sum_{i=0}^{N_{g}-1}\sum_{j=0}^{N_{g}-1}P{\left(i,j\right)}^{2}$	Std	$\sqrt{\frac{1}{n_{d}-1}\sum_{k=1}^{n_{d}}{\left(f_{k}^{\left(d\right)}-{\bar{f}}^{\left(d\right)}\right)}^{2}}$
Homogeneity	$\sum_{i=0}^{N_{g}-1}\sum_{j=0}^{N_{g}-1}\frac{P(i,j)}{1+|i-j|}$	Min	$\min_{k}f_{k}^{\left(d\right)}$
Correlation	$\frac{\sum_{i,j}\left(i-\mu_{i}\right)\left(j-\mu_{j}\right)P\left(i,j\right)}{\sigma_{i}\sigma_{j}}$	Max	$\max_{k}f_{k}^{\left(d\right)}$
Dissimilarity	$\sum_{i=0}^{N_{g}-1}\sum_{j=0}^{N_{g}-1}\left|i-j\right|\;P\left(i,j\right)$	$q_{25}$	25th percentile of $\left\{f_{k}^{\left(d\right)}\right\}$
Radius	*R*	$q_{75}$	75th percentile of $\left\{f_{k}^{\left(d\right)}\right\}$
Organoid count	$n_{d}$	Skewness	$\frac{\frac{1}{n_{d}}\sum_{k}{\left(f_{k}^{\left(d\right)}-{\bar{f}}^{\left(d\right)}\right)}^{3}}{{\mathrm{std}}^{3}}$
—	—	Kurtosis	$\frac{\frac{1}{n_{d}}\sum_{k}{\left(f_{k}^{\left(d\right)}-{\bar{f}}^{\left(d\right)}\right)}^{4}}{{\mathrm{std}}^{4}}$
**Total features (*****N*****):** 6×8+1=49			

**Table 2:** Performance of the proposed framework evaluated using stratified 8-fold cross-validation. Results are reported as mean ± standard deviation across the eight folds, with the corresponding 95% confidence intervals presented in brackets.

Target	ROC-AUC	Balanced Accuracy	F1-Score	Accuracy
TSLP	0.899±0.102	0.910±0.066	0.911±0.078	0.910±0.065
	[0.813,0.984]	[0.855,0.966]	[0.846,0.976]	[0.855,0.964]
IL-33	0.855±0.084	0.833±0.048	0.825±0.050	0.835±0.048
	[0.784,0.925]	[0.793,0.873]	[0.783,0.867]	[0.795,0.875]

**Table 3:** Performance comparison of machine learning classifiers for predicting cytokine expression (TSLP and IL-33). Metrics include accuracy (Acc), balanced accuracy (BAcc), precision (Prec), recall (Rec), and F1-score (F1), reported as mean ± standard deviation across cross-validation folds. Models evaluated include Logistic Regression (LR), Support Vector Machine with radial basis kernel (SVM-RBF), Random Forest (RF), Gradient Boosting Decision Trees (GBDT), k-Nearest Neighbors (KNN), Gaussian Naive Bayes (GNB), a Multi-Layer Perceptron (MLP), TabNet, and a convolutional neural network (CNN). The CNN baseline was trained directly on bright-field dome images, whereas the other classifiers were trained using aggregated dome-level GLCM features.

Target	Model	Acc	BAcc	Prec	Rec	F1
TSLP	LR	0.710 ± 0.179	0.705 ± 0.181	0.777 ± 0.237	0.679 ± 0.291	0.700 ± 0.230
	SVM (RBF)	0.685 ± 0.096	0.679 ± 0.083	0.785 ± 0.163	0.733 ± 0.251	0.708 ± 0.121
	RF	0.724 ± 0.137	0.728 ± 0.120	0.796 ± 0.172	0.812 ± 0.274	0.752 ± 0.143
	GBDT	0.710 ± 0.187	0.693 ± 0.179	0.745 ± 0.192	0.767 ± 0.223	0.743 ± 0.174
	KNN	0.671 ± 0.116	0.680 ± 0.111	0.733 ± 0.334	0.592 ± 0.350	0.608 ± 0.278
	GNB	0.636 ± 0.205	0.635 ± 0.190	0.630 ± 0.303	0.596 ± 0.334	0.601 ± 0.314
	MLP	0.722 ± 0.127	0.716 ± 0.128	0.755 ± 0.152	0.825 ± 0.127	0.773 ± 0.086
	CNN	0.652 ± 0.104	0.569 ± 0.154	0.321 ± 0.384	0.281 ± 0.364	0.281 ± 0.330
	**TabNet**	**0.910 ± 0.065**	**0.910 ± 0.066**	**0.961 ± 0.072**	**0.883 ± 0.145**	**0.911 ± 0.078**
IL-33	LR	0.633 ± 0.104	0.651 ± 0.076	0.604 ± 0.103	0.819 ± 0.227	0.668 ± 0.090
	SVM (RBF)	0.594 ± 0.077	0.606 ± 0.080	0.646 ± 0.226	0.738 ± 0.325	0.600 ± 0.129
	RF	0.685 ± 0.079	0.702 ± 0.079	0.612 ± 0.092	**0.976 ± 0.071**	0.745 ± 0.047
	GBDT	0.533 ± 0.113	0.534 ± 0.119	0.502 ± 0.141	0.594 ± 0.231	0.530 ± 0.149
	KNN	0.596 ± 0.088	0.592 ± 0.083	0.557 ± 0.089	0.762 ± 0.268	0.618 ± 0.136
	GNB	0.569 ± 0.088	0.589 ± 0.090	0.533 ± 0.086	0.869 ± 0.110	0.654 ± 0.061
	MLP	0.682 ± 0.091	0.693 ± 0.085	0.672 ± 0.154	0.794 ± 0.215	0.695 ± 0.083
	CNN	0.681 ± 0.135	0.684 ± 0.135	0.473 ± 0.245	0.688 ± 0.288	0.525 ± 0.175
	**TabNet**	**0.835 ± 0.048**	**0.833 ± 0.048**	**0.868 ± 0.144**	0.838 ± 0.183	**0.825 ± 0.050**

## Data Availability

The datasets used and/or analyzed during the current study are available from the corresponding authors upon reasonable request.

## Appendix A

This appendix contains the supplementary tables and figure referenced in the main text.

**Table A1:** Summary of dataset composition and class distributions used for airway organoid imaging and machine learning analysis. The table summarizes donor-wise and total numbers of HDM-stimulated Matrigel domes, bright-field images, segmented organoids, and dome-level qPCR class distributions for TSLP and IL-33 based on the predefined 1.5-fold threshold relative to untreated controls.

Category	Donor 1	Donor 2	Total
Donors	1	1	2
HDM-stimulated Matrigel domes	44	34	78
Bright-field images	44	34	78
Segmented organoids analyzed	7931	7141	15,072
TSLP (+)	27	18	45
TSLP (−)	17	16	33
IL-33 (+)	23	14	37
IL-33 (−)	21	20	41

**Table A2:** Quantitative assessment of differentiation marker variability across airway organoids. Summary statistics including sample size (*n*), mean, standard deviation (SD), and coefficient of variation (CV).

Marker	*n*	Mean	SD	CV
KRT5	16	0.151	0.111	0.735
Ac-Tubulin	16	0.073	0.074	1.006
MUC5AC	13	0.321	0.194	0.605

**Table A3:** Sensitivity analysis of qPCR cutoff thresholds for binary label definition. Performance is reported as mean ± standard deviation across cross-validation runs.

Target	qPCR Cutoff	Positive Domes	Negative Domes	Acc	BAcc	AUC	F1
TSLP	1.2	57	21	0.897 ± 0.096	0.931 ± 0.064	0.920 ± 0.083	0.921 ± 0.074
	1.3	53	25	0.883 ± 0.068	0.874 ± 0.075	0.888 ± 0.086	0.910 ± 0.059
	1.4	51	27	0.869 ± 0.112	0.895 ± 0.085	0.911 ± 0.092	0.882 ± 0.112
	1.5	45	33	0.910 ± 0.065	0.910 ± 0.066	0.899 ± 0.102	0.911 ± 0.078
	1.6	40	38	0.819 ± 0.078	0.825 ± 0.071	0.841 ± 0.089	0.812 ± 0.112
	1.7	40	38	0.819 ± 0.078	0.825 ± 0.071	0.841 ± 0.089	0.812 ± 0.112
	1.8	35	43	0.874 ± 0.103	0.880 ± 0.102	0.871 ± 0.128	0.869 ± 0.096
	2.0	28	50	0.897 ± 0.080	0.875 ± 0.107	0.860 ± 0.140	0.832 ± 0.160
IL-33	1.2	53	25	0.899 ± 0.093	0.903 ± 0.094	0.919 ± 0.079	0.921 ± 0.074
	1.3	46	32	0.869 ± 0.094	0.874 ± 0.096	0.844 ± 0.132	0.879 ± 0.091
	1.4	41	37	0.835 ± 0.090	0.840 ± 0.093	0.892 ± 0.104	0.852 ± 0.069
	1.5	37	41	0.835 ± 0.048	0.833 ± 0.048	0.855 ± 0.084	0.825 ± 0.050
	1.6	34	44	0.869 ± 0.078	0.881 ± 0.074	0.884 ± 0.107	0.872 ± 0.070
	1.7	30	48	0.822 ± 0.086	0.849 ± 0.070	0.863 ± 0.080	0.814 ± 0.062
	1.8	28	50	0.833 ± 0.092	0.843 ± 0.083	0.846 ± 0.101	0.792 ± 0.105
	2.0	23	55	0.871 ± 0.074	0.887 ± 0.059	0.906 ± 0.075	0.810 ± 0.099

**Table A4:** Hyperparameter configurations of baseline classifiers used for comparison with TabNet. All models were evaluated under the same 8-fold stratified cross-validation protocol and the same validation-based threshold selection procedure. Fixed and commonly used hyperparameter settings were adopted for each baseline model.

Model	Hyperparameter	Value
Logistic Regression (LR)	max_iter	5000
	class_weight	balanced
	solver	lbfgs
SVM (RBF kernel)	*C*	1.0
	gamma	scale
	probability	True
	class_weight	balanced
Random Forest (RF)	n_estimators	400
	max_depth	None
	min_samples_split	2
	class_weight	balanced_subsample
Gradient Boosting (GBDT)	n_estimators	300
	learning_rate	0.05
	max_depth	3
	subsample	0.9
*k*-Nearest Neighbors (KNN)	n_neighbors	5
	weights	distance
Gaussian Naïve Bayes (GNB)	priors	None
	var_smoothing	$1\times 10^{-9}$
Multi-Layer Perceptron (MLP)	hidden layers	2×256 units
	dropout	0.30
	batch normalization	enabled
	learning rate	$1\times 10^{-3}$
	weight decay	$1\times 10^{-4}$
	epochs (patience)	2000 (150)
	batch_size	64

**Table A5:** Paired Wilcoxon signed-rank tests comparing TabNet with conventional machine-learning models using fold-wise cross-validation results. Values are raw two-sided *p*-values.

Baseline	TSLP				IL-33			
	AUC	BAcc	F1	Acc	AUC	BAcc	F1	Acc
LR	0.008	0.008	0.016	0.018	0.023	0.027	0.028	0.027
SVM	0.008	0.008	0.008	0.008	0.008	0.008	0.008	0.008
RF	0.008	0.008	0.008	0.008	0.023	0.028	0.063	0.028
GBDT	0.023	0.018	0.039	0.018	0.016	0.018	0.008	0.018
KNN	0.008	0.008	0.008	0.008	0.027	0.008	0.016	0.008
GNB	0.008	0.008	0.008	0.008	0.039	0.018	0.008	0.017
MLP	0.023	0.016	0.055	0.023	0.039	0.028	0.018	0.028
